# Erythrocyte membrane protein band 4.1-like 3 inhibits osteosarcoma cell invasion through regulation of Snai1-induced epithelial-to-mesenchymal transition

**DOI:** 10.18632/aging.202158

**Published:** 2020-12-11

**Authors:** Xiaofeng Yuan, Lianhua Piao, Luhui Wang, Xu Han, Lei Tong, Shijie Shao, Xiaoshuang Xu, Ming Zhuang, Zhiwei Liu

**Affiliations:** 1 Department of Orthopedics, The Third Affiliated Hospital of Soochow University, Changzhou 213000, Jiangsu, P.R. China; 2 Institute of Bioinformatics and Medical Engineering, Jiangsu University of Technology, Changzhou 213000, Jiangsu, P.R. China; 3 Department of Urology, The Third Affiliated Hospital of Soochow University, Changzhou 213000, Jiangsu, P.R. China

**Keywords:** 4.1B, EPB41L3, osteosarcoma, Snai1, EMT

## Abstract

Erythrocyte membrane protein band 4.1-like 3 (EPB41L3) is an important membrane skeletal protein that may interact with numerous membrane proteins. Loss of EPB41L3 is reported in multiple cancer types, and it is originally identified as a tumor suppressor. In this study, through analyzing expression profiling retrieved from the Gene Expression Omnibus (GEO) dataset, we find that EPB41L3 is upregulated in primary osteosarcoma (OS) and osteosarcoma cell lines. Importantly, EPB41L3 may promote osteosarcoma cell proliferation and suppress osteosarcoma cell migration and invasion. Reduced EPB41L3 leads to a decrease of E-cadherin as well as an increase of N-cadherin and Vimentin, implying a prominent epithelial-to-mesenchymal transition. Furthermore, we demonstrate that EPB41L3 inhibits the epithelial-to-mesenchymal transition through destabilizing the Snai1 protein, one of the most important transcription factors of the epithelial-to-mesenchymal transition process. Collectively, our study has first established the complex and vital roles of EPB41L3 and implicated EPB41L3 as a potential biomarker in osteosarcoma.

## INTRODUCTION

Osteosarcoma (OS) is one of the most common primary malignancies of bone particularly with a high incidence rate in children and young adults [1]. It is characterized by the existence of malignant mesenchymal cells that produce osteoid. At present, the 5-year overall survival rate for patients with non-metastatic osteosarcoma is 60-70%, whereas the survival rate for patients with metastatic osteosarcoma is 20-30% [2]. Osteosarcoma pulmonary metastasis which precedes resection of the primary tumor is the main reason for the poor prognosis of OS patients [3]. What’s more, micro-metastasis which is estimated to be present in 60% of OS patients without obvious pulmonary metastasis cannot be effectively monitored at present [4]. The combination of neoadjuvant chemotherapy and postoperative adjuvant chemotherapy with surgical resection has improved the long-term survival rate of osteosarcoma patients. Nevertheless, patients resistant to drug treatment or intolerant to chemotherapeutic agents still present a high risk of local recurrence or distant metastasis [5]. Therefore, there is an urgent need to identify the molecular determinants governing osteosarcoma progression and novel targetable agents as adjuvant drugs for chemotherapy to improve the survival of osteosarcoma patients.

EPB41L3, a member of the FERM domain-containing 4.1 family, is identified to play crucial roles in cytoskeleton-associated processes including cytoskeletal rearrangement, intracellular transport, and signal transduction [6, 7]. The reduced EPB41L3 presumably caused by aberrant DNA methylation and/or LOH (loss of heterozygosity) is likely to be a key event in several solid tumors including lung adenocarcinoma [8–11], meningioma [12–15], breast [16, 17], ovarian [18], and prostate cancer [19, 20]. Moreover, several studies imply EPB41L3 as a potential biomarker for the diagnosis and prognosis of cervical cancer [21–26]. EPB41L3 is an important component of the membrane-associated cytoskeleton, which contributes to cell shape, cell adhesion, and cell motility. Loss of EPB41L3 may result in disrupting intercellular adhesion, loss of polarity, increased individual motility, and eventual metastasis of cancer. Prior studies have implicated that EPB41L3 can attenuate epithelial-mesenchymal transition (EMT) and metastasis by suppressing heat shock protein 5 (HSPA5) in non-small cell lung cancer (NSCLC) [27, 28]. Besides, EPB41L3 can directly inhibit the EMT process and malignancy in melanoma cells [29]. As far as we know, there is no clarity regarding the involvement of EPB41L3 in osteosarcoma, especially the EMT process, and the role of EPB41L3 in osteosarcoma remains to be explored.

The spread of cancer cells to distant organs represents a major clinical challenge to the treatment of osteosarcoma. EMT is a highly dynamic biological process in embryonic development and tissue repair and is also increasingly identified to be associated with tumor metastasis and malignancy [30]. A feature of EMT is the loss of the epithelial marker E-cadherin and β-catenin, together with the acquisition of mesenchymal markers including N-cadherin and Vimentin [31]. Transcription factors (TFs), including zinc-finger-binding transcription factors Snai1 and Slug (also named as Snai2), the basic helix-loop-helix (bHLH) factors Twist1 and Twist2, and the zinc-finger E-box-binding homeobox factors ZEB1 and ZEB2, have been identified to mediate the EMT and also contribute to tumor formation, invasiveness, and metastasis in osteosarcoma [32, 33].

In the present study, we find the upregulation of EPB41L3 expression in a large cohort of human osteosarcoma tissues and cancer cell lines. Deletion of EPB41L3 suppresses cell growth in OS, and intriguingly, reduction of EPB41L3 expression stabilizes Snai1 protein, and in turn, promotes osteosarcoma invasion and metastasis through activation of Snai1-induced EMT. Altogether, these results uncover a dual function of EPB41L3 in OS progression and elucidate a metastasis suppressive function of EPB41L3 by decreasing the stability of Snai1 protein in OS.

## RESULTS

### EPB41L3 expression is elevated in human osteosarcoma tissues and cell lines

**Figure 1:**
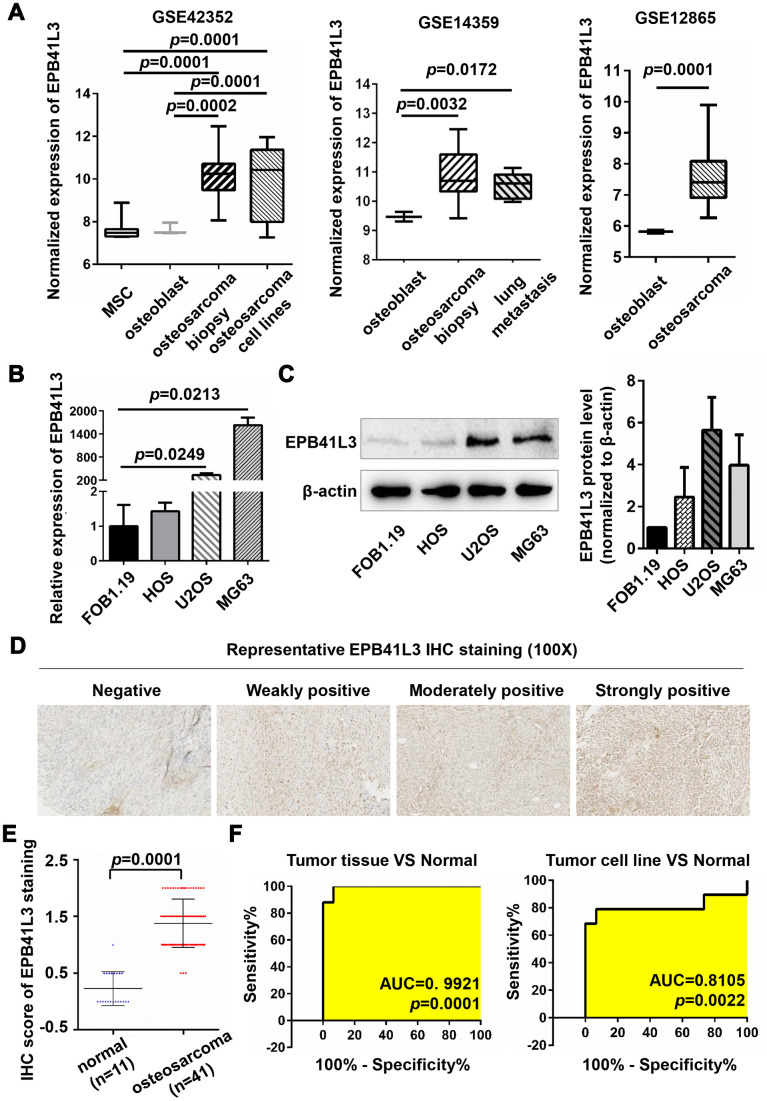
**EPB41L3 is up-regulated in osteosarcoma tissues and cell lines.** (**A**) EPB41L3 mRNA expression was significantly higher in osteosarcoma tissues and cell lines than that in normal MSC and osteoblast cells based on data from the Gene Expression Omnibus (GEO accession: GSE42352, GSE14359, and GSE12865). (**B**) Compared with normal osteoblast cells (hFOB1.19), EPB41L3 mRNA expression was significantly higher in U2OS and MG63 and not obviously up-regulated in HOS as detected by qRT-PCR. GAPDH served as the loading control and was used to normalize the expression data. Data were presented as the mean ± standard error of the mean of two independent experiments. (**C**) Western blot results showed that EPB41L3 protein expression in U2OS and MG63 but not HOS was significantly higher than that in hFOB1.19 cell. β-actin was used as a loading control and for normalization. Data were presented as the mean ± standard deviation of two independent experiments. (**D**) IHC staining results of normal bone tissues and osteosarcoma tissues (magnification, x100). (**E**) IHC total score of EPB41L3 staining was analyzed between normal bone tissues (blue scatter plot, n=11) and osteosarcoma tissues (red scatter plot, n=41). Black solid lines represented the mean ± SD. (**F**) ROC curves and AUC values for osteosarcoma based on GSE42352. EPB41L3, erythrocyte membrane protein band 4.1-like 3; MSC, mesenchymal stem cell; qRT-PCR, quantitative reverse transcription polymerase chain reaction; GAPDH, glyceraldehyde-3-phosphate dehydrogenase; IHC, immunohistochemistry; ROC, receiver operating characteristic; AUC, area under curve.

To evaluate the expression of EPB41L3 in osteosarcoma, we analyzed microarray data of the Gene Expression Omnibus (GEO) dataset (Access id: GSE42352) and found that EPB41L3 was significantly increased in osteosarcoma tissues (versus normal mesenchymal stem cells (MSCs), fold change=5.8245, *p*=0.0001; versus osteoblast cells (OBs), fold change=5.6668, *p*=0.0002) (Figure 1A). In addition, EPB41L3 expression was also elevated in OS cell lines (versus MSCs, fold change=4.8377, *p*=0.0001; versus OBs, fold change=4.7067, *p*=0.0001) (Figure 1A). Consistently, enhanced EPB41L3 expression was also observed in osteosarcoma tissues (versus OBs in GSE14359, fold change=2.5845, *p*=0.0032; versus OBs in GSE12865, fold change=3.6075, *p*=0.0001) and osteosarcoma lung metastasis tissues (versus OBs in GSE14359, fold change=2.0996, *p*=0.0172) (Figure 1A). Next, EPB41L3 mRNA and protein expression were examined through qRT-PCR and western blotting in three osteosarcoma cell lines (HOS, U2OS, and MG63) compared with normal osteoblast cell line hFOB1.19 (Figure 1B, 1C). Of those, EPB41L3 mRNA and protein expression levels were markedly higher in U2OS and MG63 compared with those in hFOB1.19, whereas slightly higher in HOS. At the same time, we analyzed EPB41L3 expression levels in 52 specimens including 11 normal bone tissues and 41 osteosarcoma tissues by immunohistochemistry (Figure 1D, 1E). Figure 1D exhibited representative results of IHC applying a score of -1 to +2. IHC analysis revealed weak or moderate (≤+1) staining of EPB41L3 in all of the normal samples (11 in 11), whereas 36.59% (15 in 41) in OS cases. Approximately 19.51% (8 in 41) of OS cases expressed EPB41L3 at strong levels (+2), and 43.90% (18 in 41) of OS cases between moderate and strong levels (Figure 1E), indicating the increased level of EPB41L3 in OS. In particular, higher expression of EPB41L3 was more likely to occur in OS patients with a higher pathology grade (G3) and TNM stage (stage IIA-IVB) (Table 1, *p*=0.0506), implying that enhanced EPB41L3 was correlated with advanced tumor stages in osteosarcoma. The expression profile dataset GSE42352 was used to calculate the ROC (receiver operating characteristic) curve. ROC analysis demonstrated that the expression of EPB41L3 can be used as a biomarker to discriminate between normal and osteosarcoma both in tissues and cell lines (Figure 1F). Previous studies implicated EPB41L3 as a tumor suppressor with reduced expression in numerous tumors including lung cancer and breast cancer. Intriguingly, our results showed enhanced expression of EPB41L3 in OS, indicating the complicated functions of EPB41L3 in carcinogenesis. Our findings illustrated the possibilities that EPB41L3 may have a specific oncogenic role in osteosarcoma.

**Table 1:** Correlation between clinicopathological characteristics and EPB41L3 expression in OS.

**Characteristics**	**Low (n=34)**	**High (n=46)**	**Total**	***p*-value**
Age				
≤14 and >65	6	8	14	0.9763
>14 and ≤65	28	38	68	
Gender				
Male	22	30	54	0.9622
Female	12	16	28	
Pathology grade				
G1-G2	23	21	44	0.0506
G3	11	25	36	
Tumor infiltration depth				
T1	4	10	14	0.2458
≥T2	30	36	68	
Stage
IA-IB (low grade)	23	21	44	0.0506
IIA-IVB (high grade)	11	25	36	

### EPB41L3 attenuates osteosarcoma cell viability

**Figure 2:**
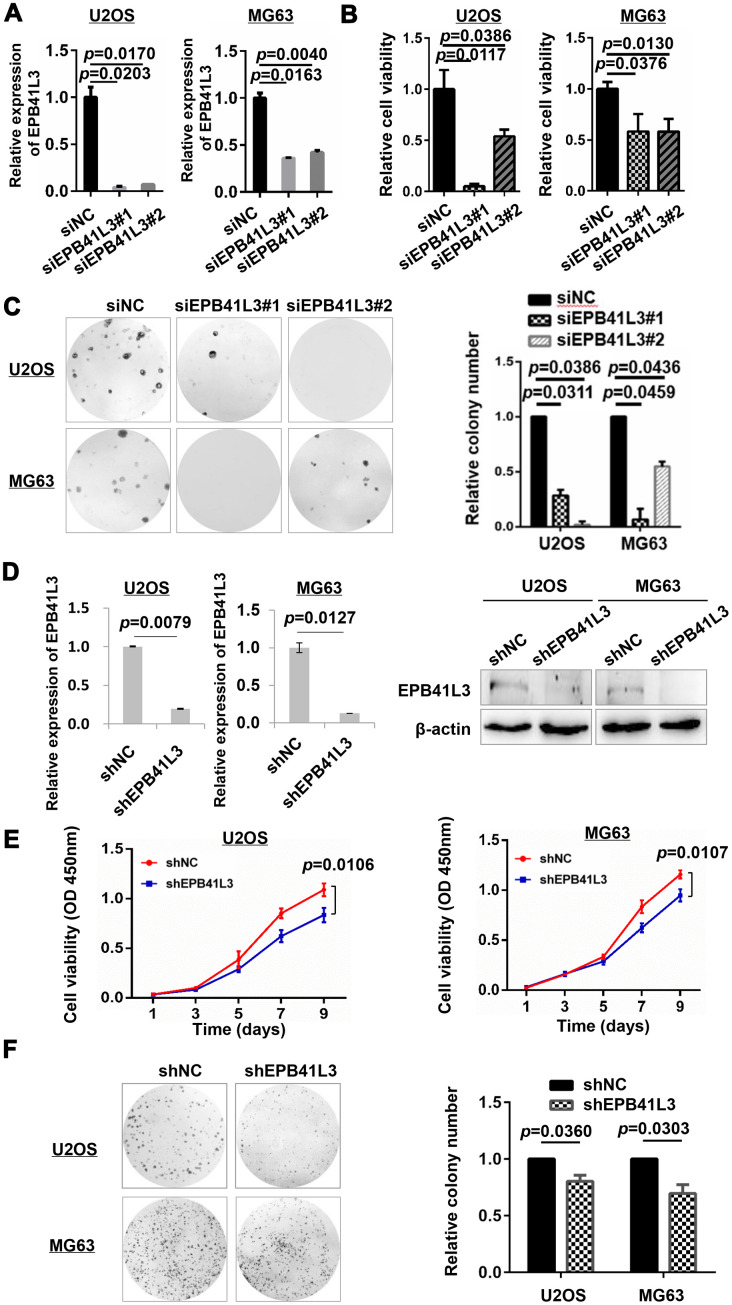
**EPB41L3 knockdown decreases osteosarcoma cell viability.** (**A**) Knockdown efficiency of EPB41L3 siRNAs (siEPB41L3#1 and siEPB41L3#2) versus control siRNA (siNC) was examined by qRT-PCR. Data were presented as the mean ± standard error of the mean of two independent experiments. (**B**) U2OS and MG63 were transfected with siEPB41L3 (siEPB41L3#1 or siEPB41L3#2) or control siRNA (siNC), and the cell viability was determined by CCK-8 assay assessed at 96 h post-transfection. Data were presented as the mean ± SD of three experimental repeats. (**C**) Colony formation assays for the effects of EPB41L3 silence in U2OS and MG63. Statistical results of colony formation numbers normalized to siNC were presented. (**D**) The efficiency of knocking down by lentiviral delivery of shRNA (shEPB41L3) was assayed by qRT-PCR (left panel) and western blot (right panel). (**E**) CCK-8 assays for the effects of stably expressing shNC and shEPB41L3 on the proliferation of U2OS and MG63 cells. (**F**) Colony formation assays for the effects of stably expressing shNC and shEPB41L3 in U2OS and MG63. Statistical results of colony formation numbers normalized to shNC were presented. NC, negative control; si, small interfering RNA.

To further examine the biological functions of EPB41L3 in human osteosarcoma, the expression of EPB41L3 was knocked down using two EPB41L3-specific siRNAs (siEPB41L3#1 or siEPB41L3#2) in U2OS and MG63 cells respectively, and knockdown efficiency was confirmed by qRT-PCR at 72 h post-transfection. Depletion of EPB41L3 was confirmed in siEPB41L3#1 or siEPB41L3#2 compared with the expression levels in siNC-transfected control cells (Figure 2A). The viabilities of siEPB41L3 or siNC-transfected osteosarcoma cells were measured by CCK-8 at 96 h post-transfection. As a result, this knockdown of EPB41L3 resulted in a drastic decrease in cell viability (Figure 2B). Colony formation assays were performed to assess the roles of EPB41L3 on the proliferation of osteosarcoma cells. Fourteen days after transfection, siEPB41L3#1- or siEPB41L3#2- transfected cells formed notably fewer and smaller colonies compared with siNC-treated cells (Figure 2C). Next, EPB41L3 stable knockdown cells were constructed using shRNA in U2OS and MG63 cells (Figure 2D), and the cell growth (Figure 2E) and clonogenicity (Figure 2F) were examined using stable cells. As shown in Figure 2E, EPB41L3 stably deleted cells exhibited a reduced growth rate compared with control stable cells in U2OS and MG63 cells. Concordantly, EPB41L3-deleted stable cells formed smaller colonies compared with control cells (Figure 2F). Together, these results also implied oncogenic functions of EPB41L3 in osteosarcoma cells.

### Downstream genes of EPB41L3 were identified by RNA-seq analysis

**Figure 3:**
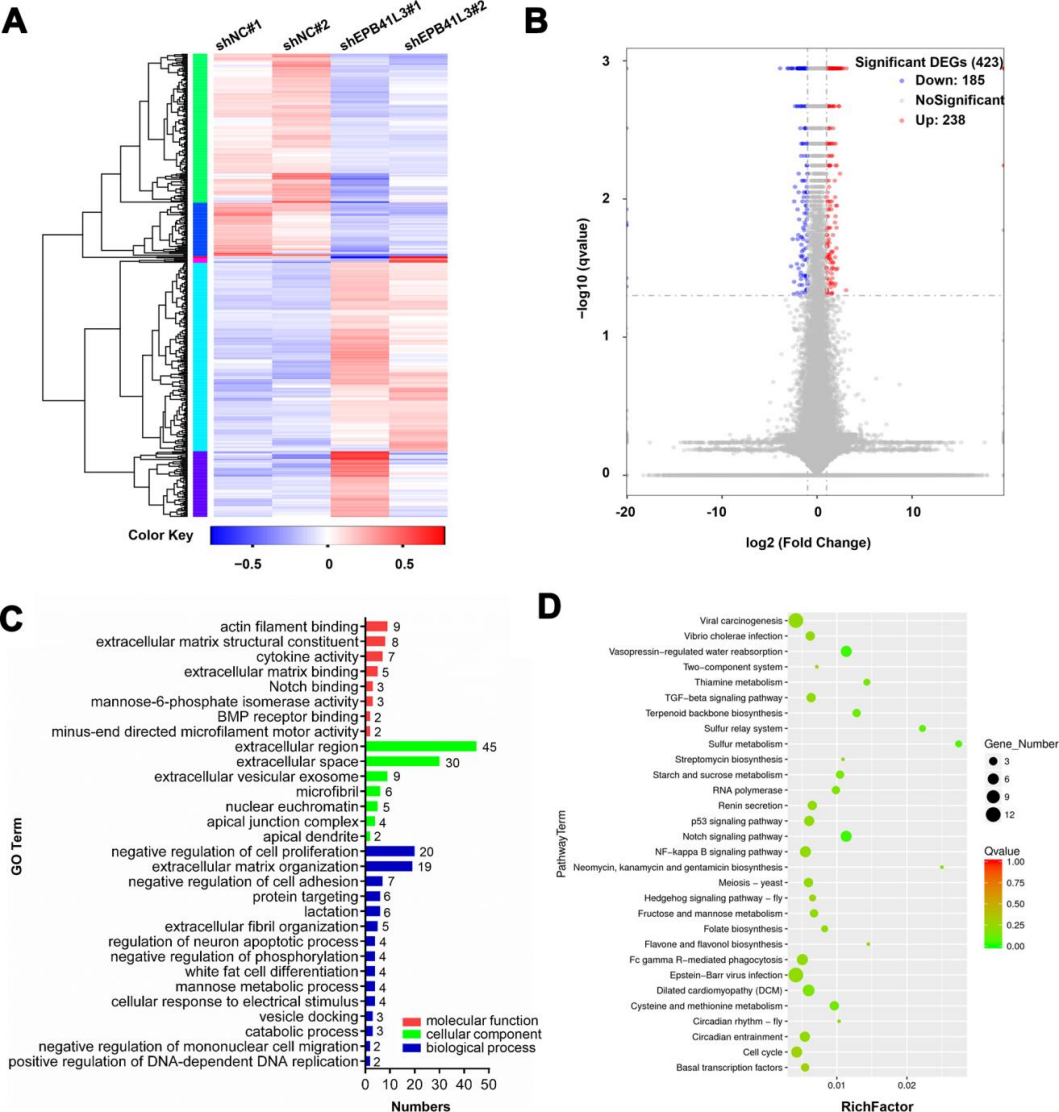
**Downstream genes of EPB41L3 are identified through RNA-seq.** (**A**) Heatmap of DEGs following depletion of EPB41L3. RNA sequencing analysis was conducted with U2OS cells stably expressing shNC or shEPB41L3. (**B**) The volcano plot of all 423 DEGs including 185 downregulated genes (blue dots) and 238 upregulated genes (red dots) based on an adjusted *p*≤0.05 and log FC≥1. (**C**) GO enrichment analysis of DEGs consisting of biological processes, cellular component, and molecular functions, was plotted according to the gene numbers. (**D**) KEGG pathway enrichment bubble chart of DEGs. The color of bubble means the significance of the corresponding KEGG pathway (in green color, low Q-value, insignificantly; in red color, high Q-value, significantly). As well, the size of bubble means the number of DEGs in this pathway. DEGs, different expression genes; FC, fold change; GO, gene ontology; KEGG, Kyoto Encyclopedia of Genes and Genomes.

To understand how EPB41L3 contributes to the development and progression of osteosarcoma, we performed RNA sequencing gene expression analysis using U2OS cells treated with EPB41L3-specific shRNA (shEPB41L3#1) or control shRNA (shNC) in duplicates. A variety of EPB41L3-regulated genes including 238 upregulated genes and 185 downregulated genes were identified with the threshold of *p*≤0.05 and a fold-change≥2 (Figure 3A, 3B). To explore the potential biological functions of EPB41L3-regulated genes, gene ontology (GO) term analysis was carried out. GO terms for each subgroup (biological process, cellular component, and molecular function) with *p*<0.05 were selected and ranked by gene numbers. GO biological process term enrichment primarily grouped most EPB41L3-regulated genes into negative regulation of cell proliferation (GO:0008285), extracellular matrix organization (GO:0030198), and negative regulation of cell adhesion (GO:0007162). Besides, in the molecular function class, these differentially expressed genes (DEGs) were annotated to actin filament binding (GO:0051015), extracellular matrix structural constituent (GO:0005201), and cytokine activity (GO:0005125) (Figure 3C). To identify the pathways enriched in DEGs, we performed KEGG pathway analysis and set *p*<0.05 as the cutoff value. In KEGG analysis, we found that DEGs were significantly enriched in viral carcinogenesis (KO05203), Epstein-Barr virus infection (KO05169), and Notch signaling pathway (KO04330) (Figure 3D).

### EPB41L3 suppresses osteosarcoma cell migration and invasion

**Figure 4:**
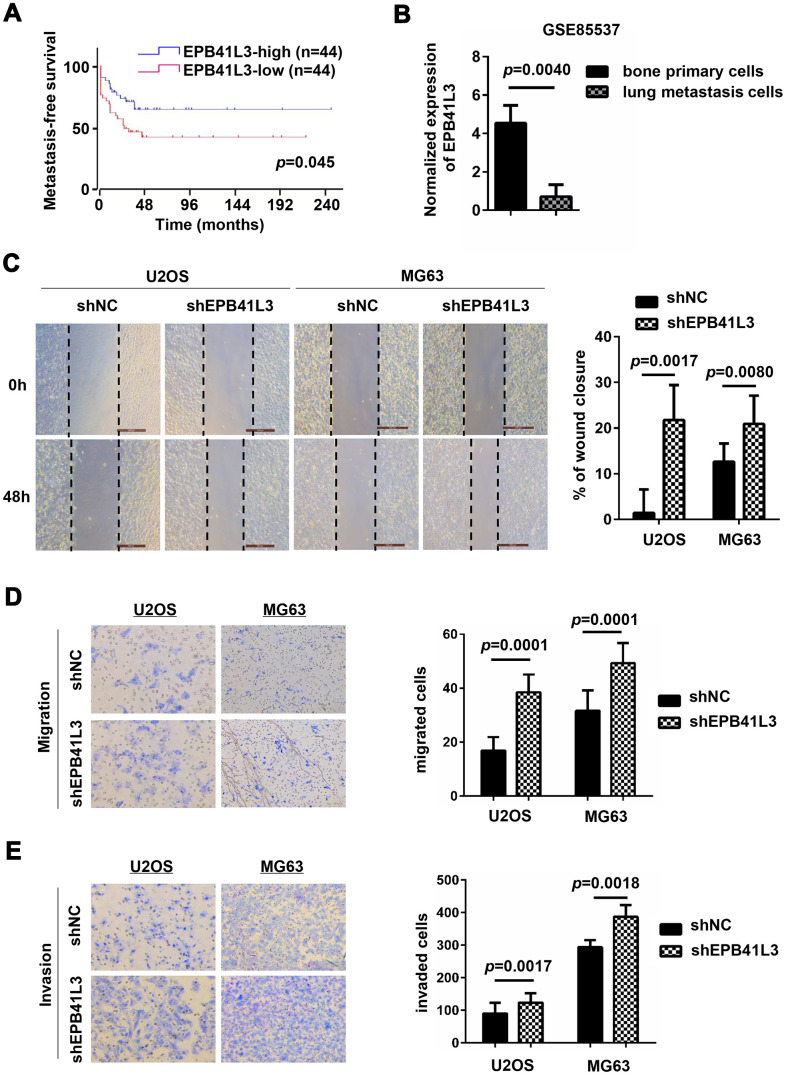
**EPB41L3 knockdown promotes migration and invasion of osteosarcoma cells.** (**A**) Metastasis-free survival analysis of patients from GSE42352 (*p*=0.0450) in the R2 online database (http://r2.amc.nl). (**B**) EPB41L3 mRNA expression was significantly higher in primary osteosarcoma cells than lung metastasis cells from GSE85537. (**C**) Wound-healing assays for U2OS and MG63 cells stably expressing shNC or shEPB41L3. (**D**) U2OS and MG63 cells stably expressing shNC or shEPB41L3 were subjected to Boyden’s chamber migration assays. Representative images of cell migration were presented (magnification, x100) and migratory cells were counted. (**E**) U2OS and MG63 cells stably expressing shNC or shEPB41L3 were subjected to Matrigel-coated invasion assays. Representative images of cell invasion were presented (magnification, x100) and invasive cells were counted. All experiments were repeated three times. Data were presented as the mean ± SD.

Previous studies have described that EPB41L3 attenuates the EMT which is considered as a critical step for metastatic dissemination in non-small cell lung cancer and melanoma [27–29, 34]. In the GSE42352 dataset, low expression of EPB41L3 had shorter metastasis-free survival (Figure 4A, *p*=0.0450). Additionally, through analyzing the GSE85537 dataset, we found that EPB41L3 expression was notably decreased in lung metastasis cells compared with primary osteosarcoma cells (Figure 4B, fold change=0.0705, *p*=0.0040). These primary findings implied the involvement of EPB41L3 in the metastatic process in OS. Hence, we examined the migratory and invasive capacities of EPB41L3 in osteosarcoma cells. Wound-healing assays showed that depletion of EPB41L3 promoted a wound closure rate compared to control cells in both U2OS and MG63 cells (Figure 4C). A Boyden’s chamber migration assay was used to measure cell migration activity of U2OS and MG63 cells stably transfected with EPB41L3 shRNA or control shRNA (Figure 4D). Similarly, Matrigel-coated invasion chambers were used to examine cell invasion activity (Figure 4E). The results showed that knockdown of EPB41L3 enhanced cell migratory and invasive capacity in U2OS and MG63 cells. These findings highlight the intriguing possibility of the involvement of EPB41L3 in osteosarcoma cell migration and invasion.

### EPB41L3 suppresses the EMT program by targeting Snai1

**Figure 5:**
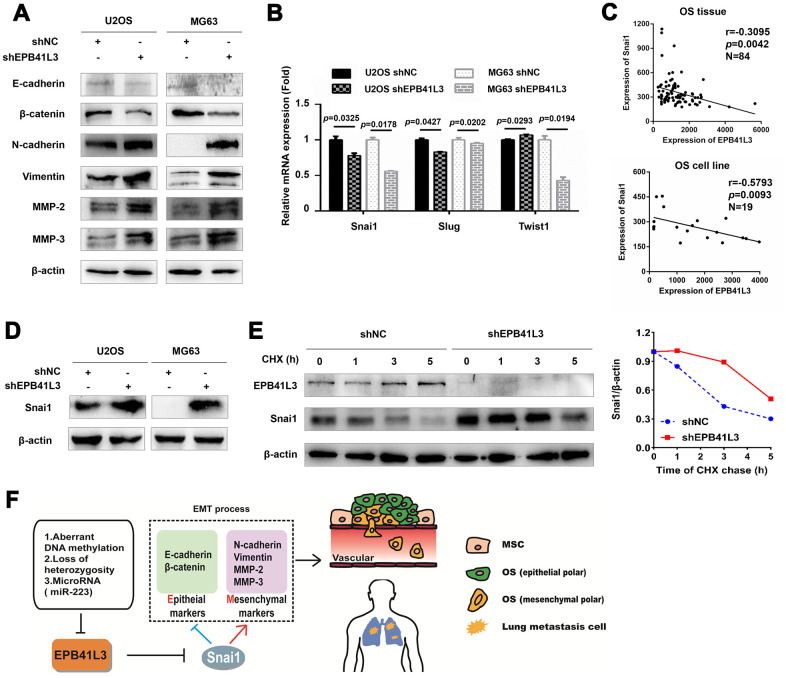
**EPB41L3 suppresses EMT and decreases the protein expression of Snai1.** (**A**) Western blot results of EMT markers in U2OS and MG63 cells stably expressing shNC or shEPB41L3. (**B**) Quantitative RT-PCR analysis suggested that EPB41L3 knockdown did not increase EMT-TFs mRNA expression including Snai1, Slug, and Twist1. (**C**) Spearman’s correlation analysis showed an inverse correlation between Snai1 and EPB41L3. (**D**) Western blot results showed that EPB41L3 knockdown increased Snai1 protein expression. (**E**) EPB41L3 knockdown enhanced Snai1 protein stability. U2OS shNC and shEPB41L3 cells were treated with 20 μM of CHX and harvested at indicated time points for immunoblotting analysis. The graph showed the relative Snai1 protein expression level (Snai1/β-actin) based on the band intensity from the gels. Snai1 protein level at 0 hour time point of CHX treatment was set as 1. (**F**) Schematic diagram of the proposed mechanism for regulatory roles of EPB41L3 on metastasis in osteosarcoma. CHX, cycloheximide.

Despite remarkable advances in the treatment of primary osteosarcoma, metastasis to distant organs is a clinically daunting event accounting for over 90% of patients. Therefore, in the present study, we focused on addressing the molecular mechanism by which EPB41L3 suppresses osteosarcoma metastasis. We found that EPB41L3-depleted U2OS and MG63 cells showed decreased epithelial markers (E-cadherin) and increased mesenchymal markers (N-cadherin and Vimentin) (Figure 5A). Besides, reduced β-catenin and enhanced MMP-2, MMP-3 was also observed upon EPB41L3 deletion. These results suggested that EPB41L3 suppresses the EMT process. EMT program is controlled by a network of transcription factors, including Snai1, Slug, ZEB1, ZEB2, and Twist1. To address how EPB41L3 is involved in the process of EMT, we next examined the effects of EPB41L3 on these EMT transcription factors. As a result, reduced mRNA levels of Snai1 were observed both in shEPB41L3-stable U2OS and MG63 cells compared to control cells, whereas slightly decreased Slug mRNA only in shEPB41L3-stable U2OS, and decreased Twist1 only in shEPB41L3-stable MG63 (Figure 5B). Spearman correlation analysis using GSE42352 showed an inverse correlation between Snai1 and EPB41L3 (r=-0.3095, *p*=0.0042 in OS tissues; r=-0.5793, *p*=0.0093 in OS cell lines, respectively) (Figure 5C). No significant correlation was found between EPB41L3 and Slug (r=-0.2731, *p*=0.0120 in OS tissues; r=0.0225, *p*=0.9272 in OS cell lines, data not shown) /Twist1 (r=-0.2799, *p*=0.0099 in OS tissues; r=0.1002, *p*=0.6833 in OS cell lines, data not shown). Although EPB41L3 was likely to positively regulate Snai1 in mRNA levels, the protein expression of Snai1 was markedly increased upon the abrogation of EPB41L3 in U2OS and MG63 cells (Figure 5D). Our findings indicated that EPB41L3 might have a more pronounced effect on Snai1 at the protein level. To understand the underlying mechanisms, we treated shNC- and shEPB41L3-stable U2OS cells with cycloheximide (CHX), a protein synthesis inhibitor. Interestingly, a gradual decreased Snai1 protein level with time was observed in CHX treated shNC-stable U2OS cells with a concomitant increased EPB41L3 protein expression. Importantly, Snai1 protein degradation was markedly slowed down in shEPB41L3-stable U2OS cells, suggesting that suppressing EPB41L3 promotes Snai1 protein stability (Figure 5E). Taken together, our results indicated that EPB41L3 suppresses the EMT program through negative regulation of Snai1 protein stability, which is specifically required for EMT initiation (Figure 5F).

## DISCUSSION

A loss of EPB41L3, presumably caused by aberrant DNA methylation and/or LOH, is frequently observed in cancer, and EPB41L3 is believed to act as a putative tumor suppressor in some cancers, primarily in NSCLC [9, 35]. To our knowledge, studies regarding the status and biological roles of EPB41L3 in human osteosarcoma have never been reported. In this study, through analyzing gene expression data from GEO series GSE42352, GSE14359, and GSE12865, we found that EPB41L3 was markedly upregulated in OS tissues and cell lines. Furthermore, we observed a significant increase of EPB41L3 mRNA and protein in OS cell lines and tumor tissues. Our findings firstly implicated entirely different roles of EPB41L3 in OS compared with other cancers. We further examined the correlation of EPB41L3 with OS clinical parameters. Notably, the IHC result showed that high expression of EPB41L3 might be correlated with high-grade OS. Besides, the silencing of EPB41L3 in two osteosarcoma cell lines suppressed cancer cell proliferation and growth. It is apparent that OS is different from other tumors in which high levels of EPB41L3 inhibits tumor progression and predicts a favorable prognosis, such as esophageal squamous cell carcinoma (ESCC) and gastric cancer (GC) [35, 36]. Moreover, prior study about gene expression profiles established from the human OS xenografts has demonstrated higher EPB41L3 expression in good responder xenografts to ifosfamide than poor responders, illustrating its important roles in the chemosensitivity of OS [37]. Therefore, identification and clarification of the biological functions of aberrant EPB41L3 expression may attribute to identifying novel targetable genes and pathways in osteosarcoma.

A key to understanding the role of EPB41L3 in osteosarcoma will be through its regulated genes. Through RNA-seq analysis, we identified a series of EPB41L3-related genes, including 185 downregulated and 238 upregulated genes upon the abrogation of EPB41L3. GO term enrichment analysis revealed that EPB41L3 was likely to be involved in cell actin filament binding, extracellular organization, cell proliferation, and cell adhesion which is one of the critical steps in tumor metastasis, indicating its specific role in OS metastasis [38].

Distant metastasis or micro-metastasis of OS at the early stage is the major cause of patients’ poor outcomes [2–4]. Recently, the EMT has emerged as a key regulator of metastasis in cancer by causing an aggressive phenotype. E- to N-cadherin switch is a significant hallmark of the EMT and often predicts poor prognosis in many cancers [39]. Recent studies illustrate that EPB41L3 is likely to be involved in the EMT process through complicated regulations of E-cadherin. A study demonstrates the interaction between E-cadherin and EPB41L3 and reveals that abrogation of EPB41L3 in immortalized mouse embryonic fibroblasts (iMEFs) causes nuclear accumulation of β-catenin [40]. EPB41L5, one of the EPB41L3 family members, destabilizes E-cadherin by binding to p120-catenin through its FERM domain [41, 42]. FRMD5 sharing the similar FERM domain with EPB41L3 interacts with p120-catenin through its C-terminal region, and this interaction reinforces the binding of p120-catenin and E-cadherin in the EMT process [43]. Consequently, it is quite comprehensible that EPB41L3 possessing similar FERM domain organization has possibilities to affect the interaction of p120-catenin with E-cadherin. A better understanding of the EMT status and the identification of appropriate molecular determinants in OS is crucial for establishing personalized treatment regimes. Our study showed that knockdown of EPB41L3 might promote cell migration and invasion through EMT in OS via enhancing Snai1 protein stability. Upon the knockdown of EPB41L3, E-cadherin and β-catenin were reduced at protein levels, accompanied by increased N-cadherin, Vimentin, MMP-2, and MMP-3. Next, we analyzed the expression of Snai1/Slug/Twist1 which was probably correlated with EPB41L3 using the GSE42352 dataset. The results showed that only Snai1, but not Slug and Twist1, was negatively correlated with EPB41L3 both in tissues and cell lines. Furthermore, knockdown of EPB41L3 had led to a remarkable increase at the Snai1 protein level. Interestingly, this enhancement of Snai1 was only detectable at the protein level but not at the mRNA level. We also found that EPB41L3 might positively regulate Snai1 in the mRNA level which was required for further investigation. Of the above, our results support a novel molecular mechanism that elucidates the role of EPB41L3 as a modulator of EMT in OS via regulation of Snai1.

The specific relationship between EPB41L3 and Snai1 remains unclear and yet to be elucidated. Snai1 is a highly unstable protein that undergoes rapid translocation between the nucleus and cytoplasm [44]. Therefore, we examined whether EPB41L3 could affect Snai1 protein stability. It is worth noting that Snai1 degradation was accelerated with a concomitant increase of EPB41L3 protein in shNC-stable U2OS cells treated with cycloheximide. Further studies have revealed that suppressing EPB41L3 may enhance Snai1 protein stability through inhibition of protein degradation. These findings indicated that EPB41L3 destabilized Snai1 protein in OS. It is still unclear how EPB41L3 destabilizes Snai1 and future studies are required to elucidate the underlying mechanisms. Although there was no significant association between Slug and EPB41L3 in the Spearman correlation study, Slug protein expression was elevated upon the silencing EPB41L3 (data not shown). These results illustrated that EPB41L3 may also modulate Slug protein which is required to be explored.

In summary, our results suggest that EPB41L3 could play both pro-tumorigenic and anti-metastatic roles in osteosarcoma, and exert its metastasis-suppressive function through inhibiting the Snai1-mediated EMT process. Our findings provide mechanistic insights into the dual roles of EPB41L3 in osteosarcoma development and progression.

## MATERIALS AND METHODS

### Microarray data collection and analysis

We screened out microarrays of OS in the Gene Expression Omnibus (http://www.ncbi.nlm.nih.gov/geo) database, accession numbers GSE42352 (included 12 normal MSC samples, 3 normal OB samples, 84 high-grade primary OS pre-chemotherapy tissue samples, and 19 high-grade primary OS cell lines), GSE14359 (included 1 normal OB sample, 5 OS tissue samples, and 4 OS lung metastasis tissue samples), GSE12865 (included 1 normal OB sample and 6 OS tissue samples), and GSE85537 (3 bone primary cell samples and 3 OS lung metastasis cell samples).

### Cell lines and small interfering RNA transfection

The 293T, hFOB1.19, HOS, U2OS, and MG63 cell lines were purchased from the Chinese Academy of Cell Resource Center, and cultured at 37° C under an atmosphere of 5% CO_2_.

To silence EPB41L3, cells were transfected with EPB41L3-specific siRNA oligonucleotide duplexes which were synthesized by Biolino Nucleic Acid Technology Co., Ltd. SiRNA against negative control was used as the control siRNA. Osteosarcoma cells with a density of 30-40% were transfected with siRNA (100 nM final concentration) using Lipofectamine® RNAiMAX (Thermo Fisher Scientific, Inc.).

### RNA extraction and quantitative real-time polymerase chain reaction (qRT-PCR)

Total RNA was isolated from 1x10^6^ cells with the TaKaRa MiniBEST Universal RNA Extraction kit (Takara Biotechnology Co., Ltd., Dalian, China). Extracted RNA was reversely transcribed to cDNA with the 1^st^ Strand cDNA Synthesis kit (Takara Biotechnology Co., Ltd., Dalian, China), according to the protocol. Quantitative real-time PCR was performed using SYBR Premix Ex Taq (Takara Biotechnology Co., Ltd., Dalian, China) and the StepOnePlus Real-Time PCR system (Applied Biosystems, shanghai, China) with the following cycling conditions: Initial denaturation at 95° C for 30 s, followed by 40 cycles of 95° C for 5 s and 60° C for 34 s. The relative gene expression levels were normalized to GAPDH and quantified using the comparative 2-ΔΔCq method. The primer sequences were listed in Table 2.

**Table 2:** Primer sequences.

**Name**		**Sequences**
EPB41L3	Forward	TCGGAGACTATGACCCAGATGA
	Reverse	GGTGCAAAGCGGAACTCACT
GAPDH	Forward	ATGGAAATCCCATCACCATCTT
	Reverse	CGCCCCACTTGATTTTGG
EPB41L3 siRNAs		
siEPB41L3#1	sense	GCACGUUCAUAACCAACAATT
	antisense	UUGUUGGUUAUGAACGUGCTT
siEPB41L3#2	sense	GGCUGCGAAUAAACAGAUUTT
	antisense	AAUCUGUUUAUUCGCAGCCTT
Negative control (siNC)	sense	UUCUCCGAACGUGUCACGUTT
	antisense	ACGUGACACGUUCGGAGAATT

### Immunohistochemistry analysis

A human osteosarcoma tissue microarray was purchased from Alenabio (Xi’an, China). For antigen retrieval, slides were immersed in 10 mM sodium citrate buffer solution (pH 6) and heated in a pressure cooker for 10 min. The slides were then incubated overnight with anti-EPB41L3 (1:100 dilution) in a moist chamber at 4° C followed by incubation for 1 h with HRP-conjugated secondary antibody at room temperature. Normal goat IgG was used as a negative control. The sections were developed with diaminobenzidine tetrahydrochloride and counter-stained with hematoxylin. IHC staining was evaluated by two independent pathologists in a blinded manner, according to the extent of positivity as follows: negative or weak EPB41L3 expression (score -1 or 0) when positive staining was present in 0-5% or 6-25% cells; moderate or strong EPB41L3 expression (score 1 or 2) when positive staining was present in 26-50% or 51-100% cells. For further analysis, scoring -1 and 0 were considered as low expression, while scoring 1 or 2 as high expression.

### Lentivirus packaging and transduction

Short hairpin RNAs targeting EPB41L3 (shEPB41L3) and negative control (shNC) were ligated into the LV-3 (pGLVH1/GFP+Puro) vector (GenePharma, Shanghai, China). The target sequences for EPB41L3 were 5’-GCACGUUCAUAACCAACAA-3’, and 5’-TTCTCCGAACGTGTCACGT-3’ as a negative control. Briefly, 293T cells were co-transfected with the lentiviral vectors LV-3-EPB41L3 (or the control lentiviral vectors) and the packaging vectors psPAX2 and pMD2G using Lipofectamine® 2000 (Thermo Fisher Scientific, Inc.). The supernatant was collected at 48 h after transfection, filtered, and stored at -80° C for subsequent assays. U2OS and MG63 cells with 40-70% confluency were incubated with the virus and 8 μg/ml polybrene for 24 h followed by subsequent selection with 2 (g/ml puromycin for 1-2 weeks. Knockdown efficiency was examined by qRT-PCR and Western blot analysis.

### RNA-sequencing (RNA-seq) analysis

The Illumina Hiseq 2000 Sequencing platform (Illumina, Inc., USA) were used in this experiment. The integrity of total RNA (extracted from U2OS cells treated with shNC or shEPB41L3) was confirmed with the Agilent 2100 Bioanalyzer (Agilent Technologies, Inc., USA). The lists of significantly differentially expressed genes (DEGs) were obtained with the thresholds of *p*≤0.05 and a fold-change≥2. The GO categories are derived from Gene Ontology (http://www.geneontology.org). Pathway analysis for DEGs was provided, based on the latest KEGG (Kyoto Encyclopedia of Genes and Genomes; https://www.genome.jp/kegg) database.

### Cell viability and colony-formation assays

The cells (1x10^4^ cells/well) transfected with siRNAs were continuously cultured for 96 h and the viable cells were measured using Cell Counting Kit-8 (CCK-8; Dojindo Molecular Technologies, Inc., China). For colony-formation assays, cells were seeded (2,000 cells/well) in 6-well plates and treated with siRNAs. After 14 days of culture, the cells were stained with 0.1% crystal violet solution. NC or EPB41L3 stably knockdown U2OS and MG63 cells (1,000 cells/well) were seeded in 6-well plates and cultured for 10 days, and subsequently stained with 0.1% crystal violet as aforementioned. Relative viable cell numbers were evaluated every two days with CCK-8.

### Migration and invasion assays

We evaluated cell migration using a scratch wound-healing assay. Cells with approximately 90-100% confluency were cultured in a 6-well plate and a scratch wound was made in the cell monolayer with a sterilized 1 ml pipette tip. The cells were washed triple with PBS to remove detached cells and incubated at 37° C in a serum-free medium. The scratch wound indicated by the reference line was imaged at 0 h and 48 h after wound by an inverted microscope, and quantified using ImageJ software. The percentage of wound closure was calculated as [1-(area of the wound at 48 hours/area of the wound at time 0)]×100%. Five random fields were selected and the mean value was calculated. For migration assay, 3x10^4^ cells in serum-free medium were seeded into upper Transwell Chambers (3464, Corning, USA) which were placed into 24-well plates with medium containing 10% FBS. After 16-20 h, the cells were washed with PBS, fixed with methanol, and stained with 0.1% crystal violet solution. Cell invasion assay was performed with Matrigel Invasion Chambers (354480, BD, USA). Briefly, 1x10^5^ cells in serum-free medium were seeded into Matrigel invasion chambers and incubated for less than 24 h at 37° C. Non-invaded cells were removed by wiping the upper side of the membrane, and the invaded cells were fixed and stained with crystal violet. Migrated cells (or invaded cells) in the randomly selected ten fields were counted under a microscope. The experiments were repeated three times.

### Western blot analysis

Cells were lysed in radioimmunoprecipitation assay buffer (RIPA) with a complete protease inhibitor cocktail (Roche Applied Science, Penzberg, Germany). The relative density of the protein band was quantified using Tanon Image Software v1.0 (Tanon Science and Technology Co., Ltd.) and normalized to β-actin band density. Anti-EPB41L3 (#154071, Abcam), anti-Vimentin (#5741, CST), anti-N-Cadherin (#13116, CST), anti-β-Catenin (#8480, CST), anti-Snai1 (#3879, CST), anti-Slug (#9585, CST), anti-E-Cadherin (#3195, CST), anti-MMP2 (#87809, CST), anti-MMP3 (#14351, CST), and anti-β-actin (#A5441, Sigma-Aldrich) were used.

### Measurement of the protein stability of Snai1

To assess Snai1 protein stability, NC, or EPB41L3 stably knockdown U2OS cells were seeded in 6-well plates for 24 h and treated with 20 μM cycloheximide (CHX, #HY-12320; MCE) for 0, 1, 3, 5 hours and harvested for Western blotting analysis. The protein bands were quantified as aforementioned.

### Analysis of osteosarcoma patients' survival

Survival data were analyzed using R2: genomics analysis and visualization platform (https://r2.amc.nl). A Kaplan-Meier curve was plotted using available clinical data from the Kuijjer et al. dataset. Tumors with gene expression levels lower or higher than the median value were classified as low or high expression status, respectively, and statistical significance in survival was calculated using the log-rank test.

### Statistical analysis

Statistical analyses were performed using SPSS version 21.0 (IBM Corp., Armonk, NY, USA). The qRT-PCR data were presented as the mean ± standard error of the mean, and the other data were presented as the mean ± standard deviation. *P*<0.05 was considered significant.
